# Identification of stable restorers and high-yielding hybrids using diverse sorghum male sterile cytoplasmic sources and established pollen parents under different water regimes

**DOI:** 10.1016/j.heliyon.2024.e39807

**Published:** 2024-10-24

**Authors:** Krishna Kasanaboina, B.V. Vara Prasad, Sonal Chavan, C.V. Sameer Kumar, D. Saida Naik, D. Srinivasa Chary, Vinod Kumar Reddy Yaram, Sunita Gorthy, Ephrem Habyarimana

**Affiliations:** a Department of Genetics and Plant Breeding, PJTAU, College of Agriculture, Rajendranagar, Hyderabad, 500030, Telangana, India; b International Crops Research Institute for the Semi-Arid Tropics (ICRISAT), 502324, Patancheru, Telangana, India; c Department of Crop Physiology, College of Agriculture, PJTAU, Jagtial, Hyderabad, 505529, Telangana, India; d Department of Statistics and Mathematics, College of Agriculture, PJTAU, Rajendranagar, Hyderabad, 500030, Telangana, India; e Department of Genetics and Plant Breeding, Naini Agricultural Institute, Sam Higginbottom University of Agriculture Technology and Sciences (SHUATS), Prayagraj, Uttar Pradesh, India; f SRTC, PJTAU, Rajendra Nagar, 500030, Tealangana, India

**Keywords:** Sorghum heterosis breeding, Cytoplasmic male sterility, Fertility restoration, GGE biplot patterns, Seed producibility

## Abstract

Sorghum hybrids demonstrated increased productivity and helped offset the decreasing cultivated areas, particularly in Asia. The diversity in cytoplasmic male sterility systems, stability of restorers and high yield of sorghum is an important factor for achieving food security and sustainability. In sorghum, hybrid production has been limited to A1 cytoplasmic source to date, primarily due to limited number of restorers on other cytoplasmic sources. This work aimed at filling this gap through diversifying and assessing the producibility and stability of cytoplasmic backgrounds across environments, evaluating the stability of restorers across cytoplasms and environments, and evaluating the performance of the hybrid F1 products under different water regimes across cropping seasons. The effects of genotypes and interactions between the genotypes and the tested environments were shown visually using GGE biplot, which also categorized the genotypes based on performance per se and stability. Analysis of variance revealed significant (P < 0.001) differences between genotypes, environments and their interaction components for all the plant characteristics studied. The various GGE biplot patterns revealed that the restorers G4 (IS 33844-5), G5 (M 35-1-19), G8 (ICSV 15014), and G10 (ICSR 13009) were stable across environments and cytoplasms. We observed the following order of superiority in cytoplasmic restoration and seed producibility, A_2_ >A_1_>A_3_ >A_4_. The A_2_ cytoplasm can therefore reliably offer seed producibility advantage to commercial seed producers. Furthermore, the which-won–where model for hybrid grain yield revealed specific adaptations of hybrid genotypes G143, G195 and G215 to first mega environment (E1 and E3), whereas G167, G110, and G112 were adapted to the second mega-environment (E2 and E4). With a GGE biplot goodness of fit of 70 % the best possible yield- and stability-based ranking of hybrid genotypes was G143, G215, G189, G200, G69, G162, G119, G54, and G81. The newly uncovered hybrid products should therefore be considered for downstream testing stages and candidates for release and commercial cultivation. Clearly, this work resulted in novel findings particularly in terms of seed producibility, universal restorers across the 4 cytoplasms and environments, and superior and stable hybrid products, all of which are expected to add value to conventional and heterosis breeding in the process of sorghum cultivar development.

## 1 Introduction

Sorghum [*Sorghum bicolor* (L.) Moench] is the world's fifth most important crop, grown extensively in the dry and semi-arid tropics [1]. It is an important staple crop for increasing food security around the world due to its widespread cultivation, resistance to both biotic and abiotic stresses, responsiveness to diverse environments, low agricultural input requirements, and utilization as a functional food with good nutritional value and a high content of health-promoting compounds [[2], [3], [4]]. Following the commercialization of the cytoplasmic-nuclear male sterility mechanism for the development of the first commercial sorghum hybrid- CSH1, a number of hybrids have been released/marketed for commercial cultivation in all sorghum growing regions across the world. In many nations, the sorghum hybrids have made a substantial contribution to higher yields of grain and fodder.

Of the several CMS sources such as A1, A2, A3, and A4 systems discovered in sorghum, the A1 (milo source) has been widely exploited for hybrid development and is source for most of the commercial hybrids worldwide [[5], [6], [7], [8]]. The A1 cytoplasmic homogeneity is the major constraint to breeding heterotic hybrids as the parental lines will have a narrow genetic base and there is always a risk of epidemics outbreak as in the case of CMS-T cytoplasm in maize hybrids in 1970s [9]. Most of the published research on OPVs and hybrids has focused on those that originate mostly in the *Milo* cytoplasm i.e., A_1_ [[10], [11], [12]], but the utilization of other CMS source has been limited especially because of the paucity of restorers for other CMS systems [13]. Apart from the issues of cytoplasmic homogeneity and availability of elite restorers, the question of whether non-milo CMS systems can be economically viable is complicated by a number of factors, such as the duration of male sterility, the effect of male sterility-inducing cytoplasm on agronomic characteristics, stability of non-milo sources across different water regimes, and the availability of commercially reliable heterosis, that contribute towards the hesitation for use of non-milo male sterility sources [[13], [14], [15]].

To realize greater heterosis, future hybrid research should focus on developing a large number of genetically diverse parental lines, however, fertility restoration of cytoplasmic male sterility sources other than A1 sources is a challenging issue. The identification of restorers for diverse cytoplasmic male sterility sources such as- A_1_, A_2_, A_3_, and A_4_ cytoplasm, becomes crucial and will serve as a basis for the successful utilization of these various CMS sources to increase production while also enhancing resistance to biotic and abiotic stresses [11]. The introduction and use of a new source of male sterility and the identification of restorers will not only increases cytoplasmic diversity but also increases nuclear diversity in those cross combinations, broadening the pool of parents for creation of new hybrids.

Once a universal potential restorer has been found, it is essential for the breeder to assess the stability of restorer lines and hybrids with different cytoplasmic origins in different water regime environments, due to the changing climatic scenario to develop a stable heterotic hybrid. Globally, crops including sorghum suffer major losses in grain and stover yields due to post-flowering drought stress [[16], [17], [18]]. When there is little or no in-season rainfall, the crop typically experiences a water deficit that intensifies as the season progresses and the crop nears maturity [19]. Compared to other cereal crops, sorghum can withstand drought circumstances better [16]. However, severe grain yield and biomass losses are experienced by the crop because of drought. To fulfil food demands, crop production will need to adjust to water shortages and maintain productivity levels constantly. Exploring and establishing high-yielding, adaptable sorghum parental lines from available germplasm and their subsequent utilization in hybrid development, evaluating the performance of generated hybrids is indispensable for drought-prone environments. Most of the hybrids available in sorghum perform well in irrigated environments and very few hybrids are available for adoption in areas that are prone to drought. By careful selection of universal restorers and stable cytoplasmic male sterile lines, it is possible to sustain the sorghum production by breeding for hybrids with enhanced productivity that are stable in diverse environments. A comprehensive grasp of genotype by environment interaction not only facilitates the strategic planning of breeding programs but also enhances the efficiency in the allocation of limited resources [20]. With the critical factors in mind, the research was conducted to identify potential restorers and evaluate the stability of hybrids across varied cytoplasmic backgrounds, particularly under conditions of drought stress to identify and exploit high-yielding sorghum hybrids with diverse cytoplasmic backgrounds that demonstrate resilience to drought stress for eventual commercial cultivation.

## 2 Material and methods

### 2.1 Parental materials and establishment of L × T crosses

Twenty-three cytoplasmic male sterile seed parents distributed into A_1_, A_2_, A_3_ and A_4_ cytoplasmic backgrounds and 12 testers well established in the ICRISAT sorghum breeding, were used in this study. The selected parental genotypes were crossed according to the Line × Tester mating design developed by Ref. [21], during the *Rabi* 2020. The crosses were made by hand pollination and 241 unique crosses were successfully completed. To evaluate the hybrid products, an alpha lattice experimental design (Table 1) was set up and included 280 genotypes, of which 241 were F1 hybrids, 35 parental lines and 4 standard checks. These materials were tested under 4 environments (2 cropping seasons × 2 irrigation regimes) *i.e*., during post rainy (PR) 2021 and summer 2022 under irrigation and post flowering drought stress.

**Table 1:** Details of experimental design and layout.

Experimental item	Description
Design	Alpha lattice
Environments for yield (E)	E1: PR 2021 (drought) E2: Summer 2022 (drought) E3: PR 2021 (irrigated) E4: Summer 2022 (irrigated)
Distance between rows	60 cm
Distance between plants within a row	15 cm
Elementary plot area	2.4 m^2^
Dimension of elementary plot	2m × 2 rows
Sowing method	Seed drilling with a tractor
Number of entries	280
Number of replications	2
Number of blocks per environment	70
Number of blocks per replication in each environment	35
Number of genotypes per block	8

### 2.2 Experiments setup, phenotyping and data analysis

Seed parents, pollen parents and their F_1_ hybrids were grown in two seasons *i.e*., post rainy 2021 and summer 2022, each, under irrigation and post-anthesis drought stress, at the International Crop Research Institute for the Semi-Arid Tropics, 502324 Patancheru, Hyderabad, Telangana, India (latitude 20° 42′ 10.59″ N and longitude 76° 59’ 57.97” E), at 285 m above mean sea level. Plants in both the water regimes were raised under same field conditions till 65 Days after sowing (DAS), ensuring that the crop does not face water stress. To simulate drought-stressed condition and to stress the plants at flowering, irrigation was stopped at 65 DAS (10 days prior to anthesis) [22]. Care was taken to avoid seepage entry of water by planting 5 boarder rows along the water stressed field.

The screening procedure for the fertility restoration trait entailed conducting test crossings with a number of CMS lines and assessing the fertility of spikelets in F1 hybrids. Testers were categorized as partial restorers, partial maintainers, maintainers, or restorers, based on the male fertility/sterility of F1 plants (Table 3). Morphological investigation of restoration percentage were recorded on F1 hybrids in the three environments *i.e*., rainy 2021 (E1), post rainy 2021 irrigated (E2) and post rainy 2021 drought stress (E3). The seed setting was measured on bagged panicles on a scale from 0 (complete sterile) to 100 (complete fertility restoration).

**Table 2:** Combined analysis of variance for Fertility restoration ability across environments, cytoplasmic sources and pollen parents; and for yield and yield component traits.

Fertility restoration ability across environments, cytoplasmic sources and pollen parents			Yield and yield component traits										
					Mean sum of squares								
Source of Variation	df	Mean Sq	Source of Variation	df	DFF	MAT	NLP	PH (cm)	PL (cm)	PW (cm)	HSW (g)	PWt (t/ha)	YLD (t/ha)
**Environment**	2	61933.6**	**Environment**	3	29330.65***	35159.6***	2355.04***	542736.05***	1178.35***	90.62***	84.65***	52.6***	52.13***
**Cytoplasm**	3	59380.7**	**Genotypes**	279	88.08***	107.72***	5.31***	9237.27***	34.48***	1.03***	0.76***	3.31***	2.11***
**Pollen parent**	11	12332.3**	**Replications**	1	26.29	174.15	7.46	277.68	0.062	4.92	1.51	0.02	0.002
**Replication**	2	83.9509	**Genotype × Env**	837	24.28***	28.39***	1.86***	557.7***	3.61***	0.25***	0.12***	0.97***	0.76***
**Environment: Cytoplasm**	6	30692.2**	**Rep × Block**	68	5.14	6.57	2.05	235.5	1.37	0.24	0.07	0.42	0.2
**Cytoplasm: Pollen parent**	33	12265**	**Residuals**	1051	3.99	4.86	0.89	148.03	1.77	0.19	0.06	0.38	0.25
**Environment: Pollen parent**	22	801.726**											
**Environment: Cytoplasm: Pollen parent**	38	340.454**											
**Residuals**	3314	56.7297											

*** Significant at 0.001 per cent level, ** Significant at 0.01 per cent level, and * Significant at 0.05 per cent level.

DFF: Days to 50 % flowering, MAT: Days to maturity, PH: Plant Height, NLP: Number of leaves per plant, PL: Panicle length, PW: Panicle width, PWt: Panicle weight per hectare, HSW: 100 seed weight, YLD: Yield per hectare.

**Table 3:** Classification of number of restorers based on restoring ability across diverse cytoplasmic sources [27,28].

Category	Seed set %	No of restorers on diverse cytoplasmic sources			
		A_1_	A_2_	A_3_	A_4_
**Strong restoration**	>90 %	2	8	0	1
**High restoration**	80–90 %	8	3	5	3
**Moderate restoration**	60–80 %	2	1	0	0
**Partial restoration**	10–60 %	0	0	0	1
**Maintainer**	<10 %	0	0	7	7

Phenotyping the hybrid F1s was done on per plant or per plot basis depending upon the trait of interest. Plant height (PH), number of leaves per plant (NLP), panicle length (PL), and panicle width (PW), were determined on randomly selected five healthy plants in a plot. Days to 50 % flowering (DFF), days to maturity (MAT), hundred seed weight (HSW), panicle weight per hectare (PWt) and grain yield per hectare (YLD) were recorded on per plot basis. To determine panicle weight and grain yield, plants in the elementary plot were hand harvested at grain maturity, panicles weighed, after weighing five panicles were selected randomly from each genotype to measure the length from the base of the panicle to the tip of the top most spikelet and average expressed in centimeters. Panicle width was measured at the broadest point in centimeters. After measuring the panicle length and width, panicles were threshed to measure grain yield. Hundred seed weight was recorded in grams by counting one hundred well filled grains from each plant and weighing them after thorough drying at 12 per cent moisture content.

### 2.3 Alpha lattice design analysis

The recorded data in alpha lattice design were subjected to analysis of variance (ANOVA) as given by Ref. [23]. The linear model of observations in alpha lattice design is as follows:

$$Y_{ijk}=\mu +t_{i}+r_{j}+b_{jk}+e_{ijk}$$

Where, Y_ijk_ is the value of the observed trait for ith treatment received in the kth block within jth replicate, t_i_ is the fixed effect of the ith treatment, r_j_ is the effect of the jth replicate, b_jk_ is the effect of the kth block within the jth replicate, and e_ijk_ is an experimental error associated with the observation of the ith treatment, jth replicate and kth block.

### 2.4 GGE biplot analysis

GGE is a widely used biplot which measures GEI data to singular value decomposition (SVD) and is equal to genotype + genotype × environment [24]. The following equation served as the foundation for the GGE biplot study: ^

$$Y_{ij}=\mu +G_{i}+E_{j}+{GE}_{ij}+e_{ij}$$

Where, Yij = average phenotype for genotype i in environment j; μ = general mean; Gi = random influence of genotype i; Ej = fixed effect of environment j; and GEij = random effect of the genotype i versus environment j interaction. eij is the residual error term associated with the ij-th observation, assumed to be independently and normally distributed with a mean of zero and a constant variance.

### 2.5 Software for analysis

An open-source software R 4.2.3 were used for performing all the statistical analysis.

## 3 Results

### 3.1 Identification of restorers across diverse cytoplasmic sources

To study the fertility restoration status, the selected 12 established testers were crossed to each of the 23 male sterile lines from different origins and pertaining to A_1_, A_2_, A_3_ and A_4_ cytoplasmic backgrounds. Crosses were evaluated for restoration % during rainy 2021 (E1), post rainy 2021(irrigated) (E2) and post rainy 2021(non irrigated, post flowering drought stress) (E3). Seed set % were recorded by observing five heads from each row bagged three days before stigma emergence. Percentage of seed set were estimated by counting the number of seeds set out of the total number of spikelet per ear head [[25], [26], [27]] and mean values were subjected to combined analysis of variance across environments and cytoplasms (Table 2). A significant variation in restoring ability of pollen parents across environments and cytoplasms were observed. Similarly, we observed significant cytoplasm × environment, pollen parent × environment and pollen parent × cytoplasm interactions. Analysis of variance for yield and yield related traits were also revealed significant (P < 0.001) differences between genotypes, environments and their interaction components for all the plant characteristics studied (Table .2). The significant effects of genotypes and genotype × environment interaction justify the implementation of technique such as biplot model, GGE biplot = genotype + genotype × environment in order to determine the environmental and the genotypic behaviors allowing targeted breeding recommendations.

A total of 12 A_1_ and A_2_ restorers, 5 A_3_ restorers, and 4 A_4_ restorers were found (Table 3, Table 4). Seven testers showed maintainer reaction on A_3_ cytoplasm, and eight testers showed maintainer reaction on A_4_ cytoplasm.

**Table 4:** Mean fertility restoration % of 12 restorers on diverse cytoplasmic sources across environments.

Genotype No	Restorers	A1	A2	A3	A4
**G1**	**M35**–**1**	90.3	92.4	0	0
**G2**	**SPV 1411**	84.8	87.2	0	0
**G3**	**CSV 18R**	87.3	89.8	0	0
**G4**	**IS 338445**	87.9	92.8	86.1	89.4
**G5**	**M 35-1-19**	87.2	90.5	86.6	86.8
**G6**	**Giddi Maldandi**	89.5	92.3	0	0
**G7**	**ICSR 17008**	79.5	79.4	0	0
**G8**	**ICSV 15014**	86.9	92.3	84.8	88.6
**G9**	**Parbhani Shakti**	90.6	92.2	0	0
**G10**	**ICSR 13009**	89.3	90.6	87.5	91.7
**G11**	**ICSR 13038**	87.5	90.7	0	0
**G12**	**ICSR 13041**	75.7	85.6	83.4	11.5
	**Mean**	86.4	89.7	36	31

Genotypes were divided into various restoration categories according to the percentage of seed set [27,28]. Proportion of the restorers restoring >90 per cent fertility in F_1_ hybrids on A_2_ cytoplasm was higher as compared to A_1_, A_3_, and A_4_ (Table 3, Table 4). Out of the 12 restorers that were evaluated, 8 restorers demonstrated strong restoration ability (>90 %) on A_2_ cytoplasm, 2 restorers exhibited strong restoration ability on A_1_ cytoplasm (*Milo*), and 1 restorer depicted strong restoration ability on A_4_ cytoplasm. High restoration (80–90 %) exhibited by 8 restorers on A_1_ cytoplasm, 3 restorers on A_2_ cytoplasm, 5 restorers on A_3_ cytoplasm and 3 restorers on A_4_ cytoplasm.

### 3.2 GGE biplot patterns for fertility restoration across cytoplasmic backgrounds and environments

#### 3.2.1 Stability assessment of restorers across cytoplasms and environments through which-won-where pattern

**Fig. 1:**
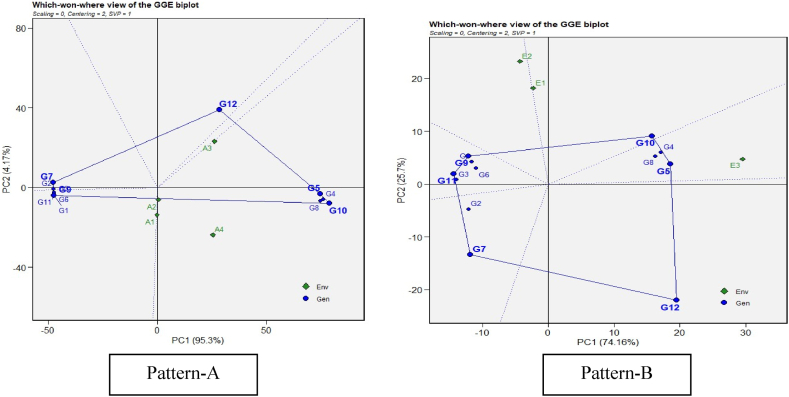
(R). “Which-won-where” pattern of GGE biplot model for restoration ability of pollen parents on A_1_, A_2_, A_3,_ and A_4_ cytoplasmic backgrounds (Pattern-A) and genotype main effect plus G × E interaction effect of sorghum genotypes in 3 three environments (Pattern-B) (Genotypes codes are in Table 4). Note: Figure No (R or H), R denotes Restorers, H denotes Hybrids.

The most effective and succinct way of summarizing the genotype and genotype × environment interaction of the dataset is the polygon view of GGE biplot which visualizes the which-won-where pattern of a multi-environment experiments [29]. The most efficient genotypes were selected based on their attributes using the GGE biplot genotype graph. The vertex genotype is the one that best adapts to a given environment or group of environments also known as mega-environment. The vertex genotype falling in the environment-free sector is expected to perform poorly across the environments. The perpendicular lines that divide the polygon into sectors are equality lines between adjacent genotypes on the polygon, and this facilitates visual comparison of them. For example, the "which-won-where" pattern analysis using different cytoplasms as testers (Fig. 1 (R), pattern A) shows that the equality line between G4, G5, G8, and G10, indicated that these four restorers performed better than the others, which performed poorly across all evaluated cytoplasmic sources. Similarly, in case of the which-won-where pattern with environments as testers (Fig. 1 (R), pattern B) demonstrates that the equality line between G4, G5, G8, and G10, indicated that these 4 restorers were better than the remaining ones that performed poorly in all the environments evaluated; however, G5 and G10 performed better in E3 (drought stress) and E1 (irrigated conditions), respectively.

The goodness of fit *i.e*., the proportion of variance explained by G + G × E was 99.47 %, of which PC1 and PC2 accounted for 95.3 % and 4.17 % respectively across A_1_, A_2_, A_3_ and A_4_ cytoplasms and 99.86 %, of which PC1 and PC2 accounted for 74.16 % and 25.7 % respectively across E1, E2 and E3 environments. If the first two principal components (PCs) explain over 60 % of the variability in the data and G and GE combined explain over 10 % of the variance, then the biplot should be able to appropriately describe the total variability in G × E data, as proposed by Ref. [30]. In the present study the total variation of the data is more than 60 %, in diverse cytoplasms and environments respectively (Fig. 1 (R). Pattern A and B), meaning that the variance explained is sufficiently high and hence the inferences derived from the GGE biplot is dependable.

#### 3.2.2 Stability assessment of restorers across cytoplasms and environments through mean vs. stability analysis

**Fig. 2:**
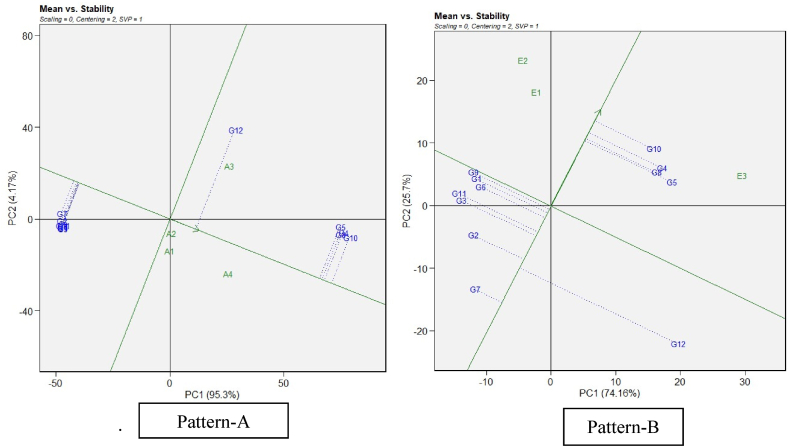
(R) GGE biplot model “mean vs stability” of sorghum genotypes restoration ability of pollen parents on A_1_, A_2_, A_3_ and A_4_, cytoplasmic backgrounds (Pattern-A) and genotype main effect plus G × E interaction effect of sorghum genotypes in 3 three environments (Pattern-B) (refer to Table 4 for genotypes codes). Note: Figure No (R or H), R denotes Restorers, H denotes Hybrids.

While the which–won-where pattern suggested winning genotypes in the specific environments, there is a need to analyze the mean performance and stability of all the tested genotypes to make selection decision. GGE biplot visualizes performance and stability graphically with the help of Average Environment Coordinates (AEC) [31]. AEC is the mean of first and second principal components scores of the test environments which is represented by the arrowhead in Fig. 2 (R). The line passing through arrowhead and origin is AEC abscissa and the line perpendicular to it at origin is ordinate. The single arrow on the first line represents the general trend towards improved performance across the environments. Length of Abscissa gives the yield of genotypes that is above-average and below-average yield depending upon whether the genotype is on the right or on the left of the origin respectively. The length of the projection of the genotypes on the ordinate is inversely proportional to the GEI (stability) associated with the genotypes; the longer the projection, the lesser the stability of the genotype of interest and vice versa [32].

Genotypes were placed in ascending order, as shown by the arrow on the AEC abscissa line, based on the cumulative value of the trait. Pattern A in Fig. 2 (R) represents the GGE biplot summarizing the mean performance and stability of restorers among cytoplasms studied, and Pattern B in Fig. 2 (R) illustrates the stability of restorers throughout the environments. Across cytoplasms, the 4 restorers (G4, G5, G8, and G10) were comparably superior to the remainders. Across environments, the same restorers confirmed their superiotrity, but, within this group G10 (ICSR 13009) showed greater and more stable restoration, while G5 (M 35-1-19) showed relatively lower stability. G7 (ICSR 17001) was the most unstable restorer. Both mean *vs.* stability GGE assessment using cytoplasms or environments as testers, were associated with more than 90 % of goodness of fit.

#### 3.2.3 Identification of best and ideal restorer genotype through genotype ranking

**Fig. 3:**
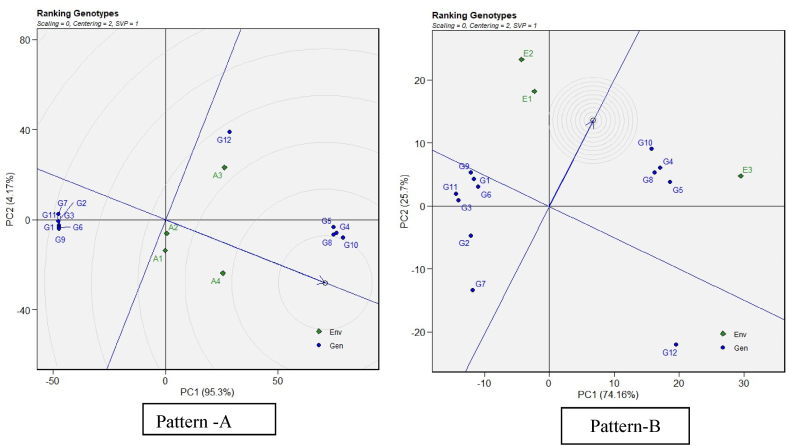
(R) The GGE biplot ‘genotypes ranking’ pattern for genotype comparison with ideal genotype showing G + G × E interaction effect on A_1_, A_2_, A_3_ and A_4_, cytoplasmic backgrounds (Pattern-A) and across 3 three environments (Pattern-B). Note: Figure No (R or H), R denotes Restorers, H denotes Hybrids.

Under GGE biplot analysis, an ideal genotype should be characterized by both high mean performance and high stability across environments. In Fig. 3 (R), the "ideal" restorer is represented at the center of the concentric circles on the average environment axis (AEA). This point is "absolutely stable" in the positive direction and has a vector length equal to the longest vectors of the restorers on the positive side of the AEA, indicating the highest mean restoration. The interpretation is that restorers located closer to the ‘ideal restorer’ are more desirable than the remainders. Thus, for the GGE across cytoplasm, the four restorers (G4, G5, G8, and G10) are equally more desirable than the rest. For the GGE across environments, the same four restorers are more desirable than the others, but G10 was more desirable among the four testers. Under GGE biplot, the stability is meaningful only when related mean performance is also meaningful. Therefore, the above four restorers (G4, G5, G8, G10) are desirable because they satisfy the requirements of stability and performance; and this was true both across cytoplasms and across environments.

#### 3.2.4 Identification of stable and high-performance restorers using best linear unbiased predictions

**Fig. 4:**
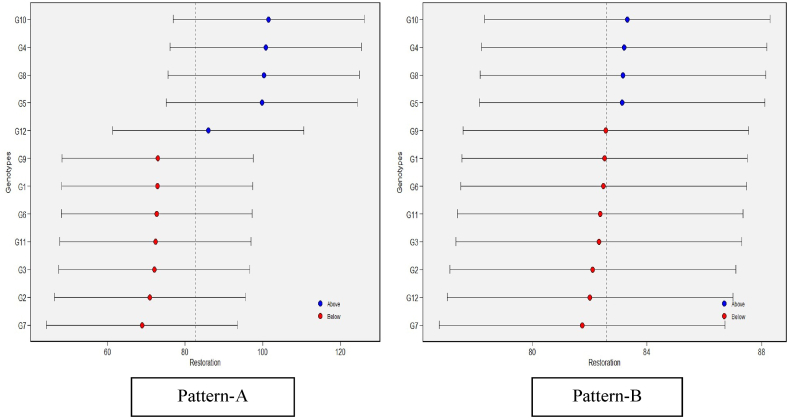
(R) Estimated values of average of the stability and mean performance for 12 restorer genotypes across cytoplasmic sources (Pattern-A) and across environments (Pattern-B) Note: Figure No (R or H), R denotes Restorers, H denotes Hybrids.

The restoration performance and the stability of the 12 restorers were evaluated across cytoplasms and across environments using linear-mixed effect models to predict the response variable (Fig. 4 (R)). The genotypes in blue circles were the best in terms of their mean performances, while the red circles illustrate the genotypes with performances below the average, and the genotypes at the bottom were the poorest. The horizontal error bars shown in Fig. 4 for each genotype denote the 95 % confidence interval for the estimated values for restoration percentage. Among the tested genotypes for restoration ability across three environments and four cytoplasms, G10 had the highest mean performance, followed by G4, G8, and G5, all of which were above average. In contrast, G9, G1, G6, G11, G3, G2, and G12 performed poorly, falling below the average. While the genotypes, G12 across cytoplasms and G7 showed the least restoration ability across cytoplasms and environments.

### 3.3 Combining ability of identified restorers

Most often per se performance of a parent is not always a true indicator of its potentiality in hybrid combination. Therefore, general combining ability, which is also the breeding value of the parent, has proved as a useful tool for choosing the parents for hybridization.

An overall assessment of GCA effects under various environments in this work i.e., post flowering drought stress, irrigated environment and pooled analysis revealed that among the testers, G9 (Parbhani Shakti) and G11 (ICSR 13038) were good general combiners for yield in all the environments (Table 5). Therefore, these parents were noted as good sources of favorable genes for increasing grain yield; using these parental lines would boost grain yield in sorghum for post flowering drought stress, as well as irrigated conditions. Among the newly identified universal restorers, G8 (ICSV 15014) and G10 (ICSR 13009) had shown excellent general combining ability in irrigated grain production environments. Furthermore genotypes, G10 and G8 showed displaying substantial levels of specific combining ability with ICSA 747 (SCA: 0.56) and ICSA 724 (SCA: 0.97) respectively.

**Table 5:** GCA effects of the restorers for yield under post flowering drought stress, irrigated and pooled environments.

S. No	Restorers	PFD	IE	PE
1	**M35**–**1**	−0.07	−0.12∗	−0.10∗∗
2	**SPV 1411**	−0.12∗	−0.20∗∗	−0.16∗∗
3	**CSV 18R**	0.02	0.08	0.04
4	**IS 338445**	0.06	−0.19∗∗	−0.07
5	**M 35-1-19**	−0.04	−0.27∗∗	−0.16∗∗
6	**Giddi Maldandi**	0.06	0.02	0.04
7	**ICSR 17008**	−0.33∗∗	−0.36∗∗	−0.35∗∗
8	**ICSV 15014**	−0.09	0.12∗	0.01
9	**Parbhani Shakti**	0.27∗∗	0.47∗∗	0.36∗∗
10	**ICSR 13009**	−0.04	0.16∗∗	0.05
11	**ICSR 13038**	0.43∗∗	0.63∗∗	0.53∗∗
12	**ICSR 13041**	−0.07	−0.09	−0.09∗

PFD-Post Flowering Drought, IE-Irrigated Environment and PE-Pooled Environment. ∗∗ Significant at 0.01 per cent level and ∗ Significant at 0.05 per cent level.

.These two universal restorers can therefore be used for several purposes including restoration across cytoplasms and environments, breeding pipelines and population improvement, and heterosis breeding.

### 3.4 Path coefficient analysis

For sorghum breeding to be as effective as it is with other crops, breeders need to be aware of the direct and indirect importance of each trait and know the direction and magnitude of the correlation between the economic (grain yield) outcome and the different component traits. The correlation coefficients between grain yield and all of the examined parameters were low, with the exception of PL, PW, and PWt. PL and PW demonstrated medium correlation, while PWt showed a significant positive association with the yield (Table 6).

**Table 6:** Phenotypic (P) path coefficients of yield and its component traits in sorghum.

	DFF	MAT	PH	NLP	PL	PW	PWt	HSW	YLD
DFF	**−3.65**	3.35	0.005	0.30	−0.001	0.00	−0.14	0.02	−0.11
MAT	−3.65	**3.35**	0.01	0.30	−0.003	−0.01	−0.13	0.02	−0.11
PH	−0.18	0.30	**0.11**	0.16	0.003	−0.34	0.54	−0.18	0.41∗∗∗
NLP	−2.88	2.68	0.04	**0.38**	0.009	−0.23	0.36	−0.06	0.30∗∗∗
PL	0.03	−0.06	0.002	0.02	**0.16**	−0.20	0.60	−0.01	0.54∗∗∗
PW	0.00	0.06	0.07	0.17	0.064	**−0.51**	1.02	−0.13	0.75∗∗∗
PWt	0.43	−0.36	0.05	0.11	0.080	−0.44	**1.20**	−0.11	0.97∗∗∗
HSW	0.51	−0.33	0.09	0.11	0.008	−0.33	0.62	**−0.21**	0.48∗∗∗

^a^The pathways are read horizontally, with each row representing a distinct path from one characteristic (exogenous variable) in the first column to the resulting plot yield (PY) via intermediate variables. direct and indirect effects are represented by diagonal and off-diagonal values in a row respectively. To make the table easier to read, we highlighted the indirect effects [33] that were larger than or equal to 0.10. The right-most column represents the Pearson's correlation coefficient between respective exogenous variable and the outcome variable, and each coefficient was decomposed into the corresponding direct and indirect effects [34]. Traits in the first column from top to bottom: DFF: Days to 50 % flowering, MAT: Days to maturity, PH: Plant Height, NLP: Number of leaves per plant, PL: Panicle length, PW: Panicle width, PWt: Panicle weight per hectare, HSW: 100 seed weight, YLD: Yield per hectare. Phenotypic Residual effect = 0.040, ∗∗∗ Significant at the P < 0.001 probability levels.

The latter three plant characteristics should therefore be considered in the indirect selection process to isolate superior sorghum genotypes with higher grain yield potential. While plant breeders often select for numerous traits, divergent genetic associations may slow advancement. Our research showed a positive relationship between three parameters and the grain yield, suggesting that tandem selection and index selection could be used to increase all attributes at once.

#### 3.4.1 Identification of stable hybrid genotypes for yield and yield components through which-won-where pattern

**Fig. 5:**
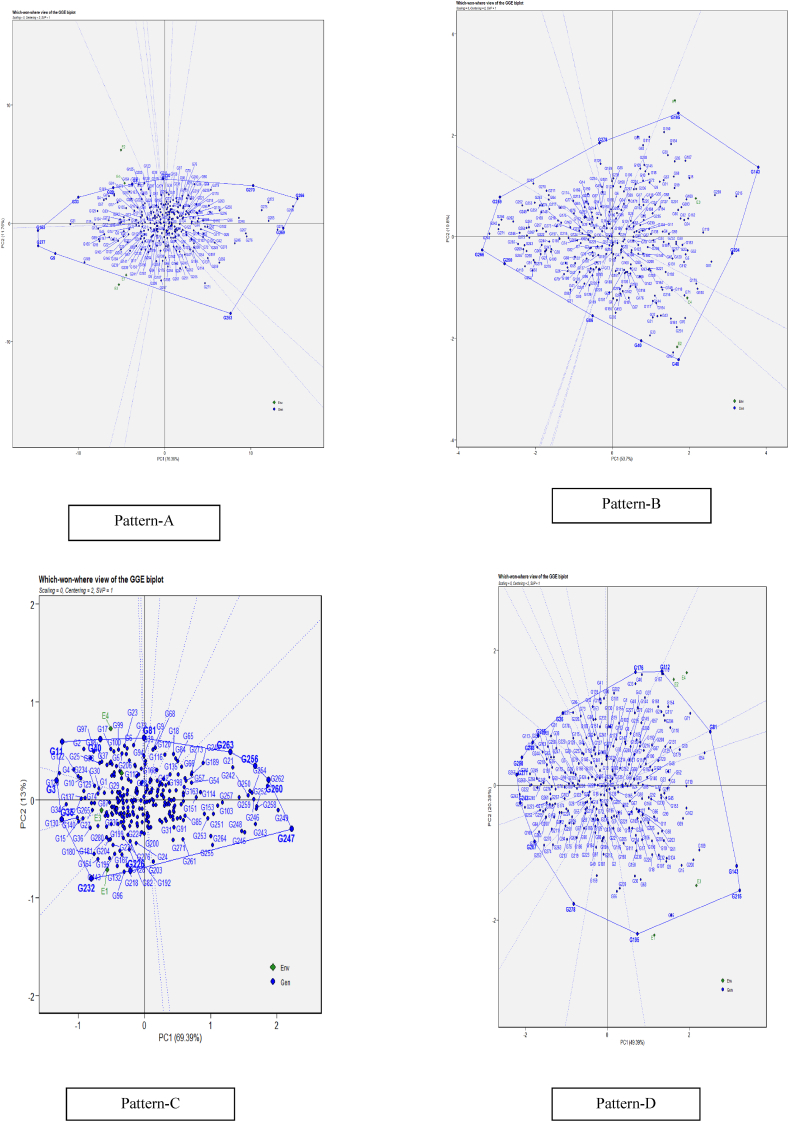
(H) GGE biplot pattern of “Which-won-where” polygon view displaying the genotype main effect plus G × E interaction effect of sorghum genotypes under four environments for panicle length (Pattern-A), panicle weight per hectare (Pattern-B), hundred seed weight (Pattern --C), grain yield per hectare (Pattern D). The biplots were created based on Centering = 2, SVP = 1 Scaling = 0. The key to the genotype labels along with mean values of four environments given in Supplementary Table 2. **Note: Figure No (R or H), R denotes Restorers, H denotes Hybrids.**

The GGE biplot of which-won-where (Fig. 5 (H): Patterns A, B, C and D) displayed that the goodness of fit (PC1 and PC2) of 91.14 %, 73.5 %, 82.39 %, 69.8 % for panicle length (Pattern A), panicle weight per hectare (Pattern B), 100 seed weight (Pattern C) and yield per hectare (Pattern D), respectively. The test environments fell into two of the twelve sectors, three of the ten sectors, three of the ten sectors, and two of the fifteen sectors outlined on the polygon view for PL, PWP, HSW, and YLD respectively. Thus, the mega environments were identified for each trait. There were two mega-environments for PL and YLD, three mega-environments for PWP and HSW. Out of the two identified mega environments for panicle length (PL), the vertex genotypes identified for the first mega environment (E1 and E3) were G9, G153, G189, and G277, while for the second mega environment (E2 and E4), were G8, G20, G33, and G99. For panicle weight per hectare, G40, G48, and G86 were identified for the first mega environment (E1 and E3), and G204, G143, and G195 for the second mega environment (E2 and E4). While for hundred seed weight, the stable and best-performing genotypes were G4, G11, and G40 in the first mega environment; G3 and G35 in E3; and G232, G96, G226, and G128 in the second mega environment (E1). The high-yielding and adaptable genotypes for the first mega environment (E1 and E3) were G143, G195, and G215.

#### 3.4.2 Assessment of stable and ideal genotypes for yield and yield components through mean *vs*. stability analysis

**Fig. 6:**
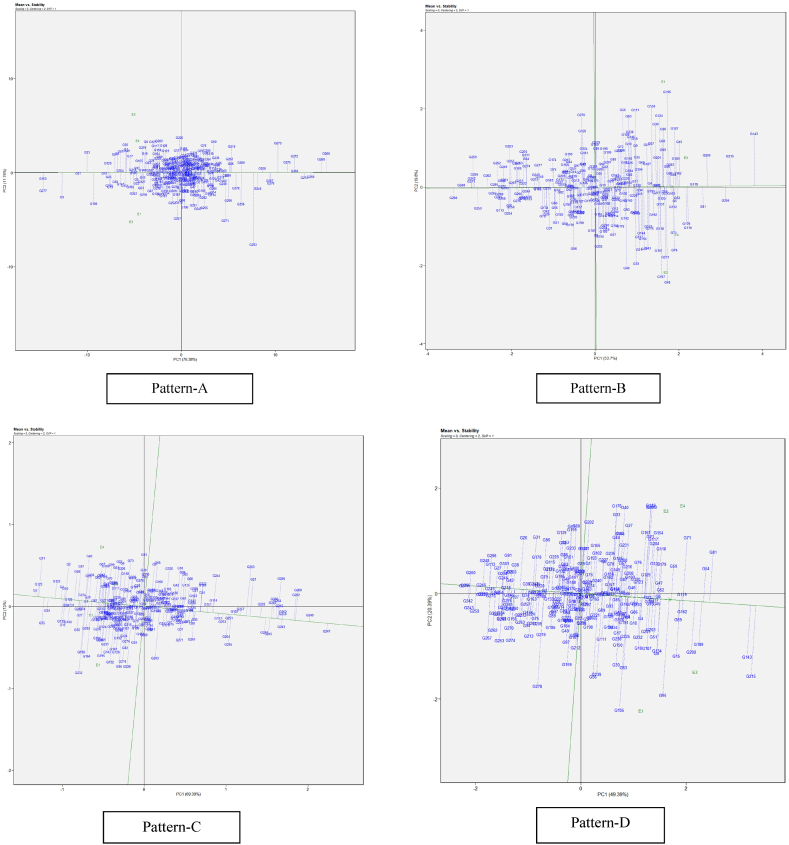
(H) GGE biplot pattern of ‘Mean vs. stability’ illustrating interaction effect of sorghum genotypes under four environments for panicle length (Pattern-A), panicle weight per hectare (Pattern-B), hundred seed weight (Pattern-C), yield per hectare (Pattern-D). The biplots were created based on Centering = 2, SVP = 2, Scaling = 0. **Note: Figure No (R or H), R denotes Restorers, H denotes Hybrids.**

The genotypes with above-average yields are located to the right of the origin, and those with below-average yields. Fig. 6 (H) represented the genotypes with above-average and below-average performance, as well as varying levels of stability. The following genotypes were yielded above the average means and exhibited higher stability, as indicated by their position toward the arrow and length of the projection for the respective traits: Panicle length (Pattern-A): G33, G153, G277, G9, G21, G33, G15, G45, G129, G165, G19, G69, G147, G151, G154, G213, G10, G93, G155, G148, G43, G5, and G22. Panicle weight per hectare (Pattern-B): G143, G215, G204, G200, G81, G119, G179, G110, G15, G162, G52, and G5. Hundred seed weight (Pattern-C): G3, G123, G35, G34, G122, G4, G10, G130, G15, G140, G125, G26, G137, G58, G74, G27, and G87. yield per hectare (Pattern-D): G143, G215, G81, G54, G189, G200, G162, G69, G15, G119, G162, G69, G74, G50, G5, G52, G47, G77, and G45. These genotypes were identified as above-average yielders with greater stability.

Based on PL, HSW, PWP and YLD, G4, G9, G10, G11, G15, G87, G71, G50, G143, G81, G200, G215, G162 and G54 were the highest performing genotypes with greater stability across all environments, while G246, G249, G14, and G266 were the lowest performing genotypes. Ideal lines have the highest yield and absolute stability lying in the arrowhead and the distance of other lines measures the desirability of lines. Based on PL, HSW, PWP and YLD G5, G50, G4, G119 and 121 had the shortest projection and considered as highly desirable genotypes for irrigated and drought stress environment, while G220, G40, G226 and G202 had the longest and considered as highly unstable and unfavorable for all the environments.

#### 3.4.3 Genotype ranking: best and ideal genotype identification

**Fig. 7:**
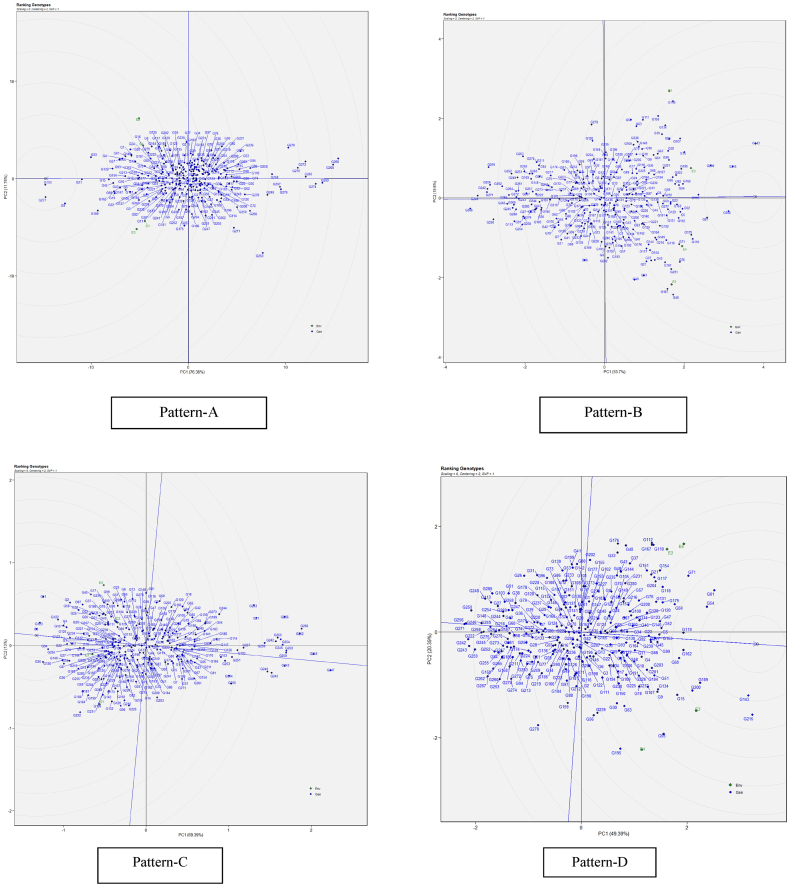
(H) The GGE biplot ‘genotypes ranking’ pattern for genotype comparison with ideal genotype showing G + G × E interaction effect of sorghum genotypes under four environments for panicle length (Pattern-A), panicle weight per hectare (Pattern-B), hundred seed weight (Pattern-C), grain yield per hectare (Pattern-D). The biplots were created based on Centering = 2, SVP = 1, Scaling = 0. The ideal genotype is signified by a circle within innermost concentric circles on average environment coordinate (AEC) abscissa which passed through biplot origin.

The following genotypes were identified as ideal and positioned towards the arrow.: G153, G277, G9, G21, G33, G189, G15, G45, G129, G165, G69 and, G19 for panicle length (Fig. 7 (H): Pattern A), G204, G215, G143, G200, G81, G119, G162, G52, G5, G15, G89 and G54 for plot panicle weight per hectare (Fig. 7 (H): Pattern B), G8, G123, G34, G35, G11, G122, G4, G10, G125, G22, G15 and G2 could be regarded as the best leading genotype for hundred seed weight (Fig. 7 (H): Pattern C), G143, G215, G81, G54, G189, G119, G162, G69, G200, G15, G71 and G50, Genotypes G71, G50, G15, G143, and G69 for yield per hectare genotype (Fig. 7 (H): Pattern D) were considered as best.

### 3.5 Seed producibility of seed parental lines

Each hybrid has a different level of producibility depending on various factors, such as the female parent's yielding ability, synchronization of female and male ﬂowering, minimal processing losses, good seed quality features, etc. The yield potential is a genetically determined ability of parents/genotypes to generate optimal yield in speciﬁc environments. A minimum seed yield of the female parent is required to reduce the cost of production. Further, as both female and male are individual inbred lines, they may respond differently to a given agroclimatic condition. Among evaluated cytoplasmic sources A2 has shown higher seed producibility with 94.5 % (Table 7) followed closely by A1 (93.7 %), A4 (92.8 %) and A3 (92.4 %).

**Table 7:** Seed producibility of seed parental lines from different Cytoplamic Backgrounds.

Sl.No	Cytoplamic Background	Mean
1	A1	93.7
2	A2	94.5
3	A3	92.4
4	A4	92.8

## 4 Discussion

To guarantee food security, there is a need to increase crop productivity through crop improvement and breeding programs. Multi-environment experiments, commonly called genotype-by-environment interactions, constitute the important steps in crop improvement and breeding programs. It is then the responsibility of breeders to increase crop productivity and make sure that the growing human population is properly fed. By the turn of the century, it is predicted that there will be more than 11 billion people on the planet, placing tremendous pressure on the world's current agricultural systems [35]. Climate change is making this more difficult by steadily reducing production and acreage. Sorghum is ideally suited for production in such regions due to its high and stable water-use efficiency, drought and heat tolerance, and extensive germplasm variability. Exploring and establishing high-yielding, adaptable sorghum parental lines from available germplasm and their subsequent utilization in hybrid development, testing the performance of generated hybrids is essential for drought-prone environments. Therefore, GGE models are employed in the current study to select genotypes with high adaptability and stability, and to identify entries with environment-specific adaptation.

In order to develop a stable heterotic hybrid, a hybrid breeder's primary responsibility is to develop and validate stable, superior parental lines from the available germplasm for various water regimes. Identifying suitable and stable restorers for alternate cytoplasmic sources within the germplasm is a significant challenge, with low chances of success. Assessing the stability of these restorers is a crucial component of hybrid or heterosis breeding programs. The success of a hybrid breeding program relies heavily on restorer lines, as they not only restore fertility but also contribute significantly to the genetic makeup of the hybrid progeny.r Identification of stable restorers helps in maintaining the desired performance and characteristics of hybrids across different environments. Restoration of fertility ensures the sustainability of hybrid seed production. Fertile restorer plants can be used as parents in subsequent breeding cycles, allowing for the continuous development of improved hybrids with desirable traits. Stable restorer parent enables the introgression of genetic diversity from diverse parental lines into hybrids, thereby enriching the gene pool, and also enhances the adaptability and resilience to changing environmental conditions. Sustainable seed production is essential for meeting the demands of modern agriculture, where high-yielding and resilient crop hybrids are needed to feed a growing global population. Stability of restorer also prevents fluctuations in hybrid performance that may arise from variability in genetic backgrounds, under different environments and it enables the breeders to select restorer lines with traits suited to specific environmental conditions or production systems. This adaptability is essential for developing hybrids resilient to climate change, and other environmental challenges, ensuring sustainable agricultural practices.

Based on the analysis of mean restoration percentages and stability across various environments and cytoplasmic backgrounds, among the tested genotypes, ICSR 13009 (G10) stands out with the highest stability and mean fertility restoration rate of 89.2 %, closely followed by IS 33844-5 (G4, 87.4 %), ICSV 15014 (G8, 87.0 %), and M 35-1-19 (G5, 86.0 %). These genotypes (Table 4 and Fig. 2 (R)), were identified as universal and stable restorers across different environmental conditions and cytoplasmic backgrounds, using different GGE biplot analytics. Further examination of mean restoration across different cytoplasmic backgrounds reveals that, A2 cytoplasmic background exhibited the highest mean percentage of fertility restoration of 89.7 %, followed by A1 (86.4 %), A3 (36 %), and A4 (31 %). The identified restorers can be utilized in hybridization programme for further understanding the cytoplasmic effect on various traits performance using iso-nuclear and alloplasmic lines. Despite sharing the same pollen parent, different cytoplasmic sources from different origins demonstrated varying degrees of success in restoring fertility. This discrepancy suggests that differences in the expression and interaction of restorer genes with the cytoplasm contributed to the observed restoration patterns in these cytoplasmic male sterile (CMS) lines. Similar findings were reported by Refs. [9,19,27]. Among the identified restorers, i.e., ICSR 13009 (G10), ICSV 15014 (G8), M35-1-19 (G5), and IS 33844-5 (G4), the latter two were previously utilized in sorghum breeding [9] but lacked empirical confirmation as universal restorers. We were therefore able to identify novel universal restorers, most of which showed good GCA and SCA. These four genotypes can be selected based on their high stability for restoration for future utilization in hybrid breeding, hold promise for the development of superior hybrids, offering valuable insights into hybrid breeding strategies for sorghum improvement.

To further understand how well genotypes perform agronomically across all of the test sites, we may use the mean vs. stability yield analysis in conjunction with the genotype ranking analysis [36]. Results manifested that the genotype, G10 had the most stable restoration ability due to its shortest projection, whereas G12 had the most unstable restoration capacity due to its longest projection. We found the best stable yield and yield component trait projections for PL, PPW, HSW, and YLD to be G15, G4, G87, and G45, respectively (Fig. 6 (H), Patterns A, B, C, D). The most unstable genotypes were determined to be G253, G48, G232, and G195 due to their longest projection. The GGE biplot uses average yield and relative stability to find the desirable genotypes. The results depicted that the genotype G45 was a stable, high-yielding, which suggested that it was well-buffered and could avoid environmental performance variations [37]. High and sustained production potential over a broad variety of environmental circumstances was defined as criteria for a successful cultivar [38]. These trends resonate with findings reported by Refs. [[39], [40], [41]], emphasizing the importance of multi-environment trials in deciphering the influence of genotype × environment interaction (GEI) on genotype performance. The interaction between genotypes and the environment indicates that selecting desired genotypes should consider not only their mean performance but also their stability to prevent significant commercial losses. Genotype G45 was identified as the most desirable in all four scenarios due to its exceptional combination of high yield and stability.

Plant breeders utilize agronomic performance data to select genotypes that thrive in specific environments, considering both mean performance and stability [42]. The ideal genotypes were those closest to the center of the concentric circles for each agronomic trait studied. Genotypes farther from the arrowhead were likely to have lower yields. In most cases, the optimal genotype is located within the innermost circle, closest to the arrowhead at the center of the ring. These inner circle genotypes are far more suitable than those in the outer circles. However, when no genotype is found within the innermost circle, those positioned just outside it are considered ideal [41,43]. Because of their stable genotypes and proximity to the biplot origin, the following were considered excellent genotypes throughout the studied environments: Based on restoration ability genotypes G10, G4, G8 and G5 (Fig. 3 (R)), these restorers are crucial for producing vigorous and high-yielding hybrids under drought stress, as they contribute complementary genetic factors that enhance hybrid vigor and stress resilience. Whereas based on PL, PPW, HSW and YLD, Genotypes G71, G50, G15, G134, and G9 (Fig. 7 (H): Pattern D) were considered as best.

According to the ideal genotype perspective, the genotypes in the first three circles near the concentric circle for traits such as panicle weight per hectare and yield per hectare were nearly identical and could be considered the most likely ideal genotypes. Their consistent and stable performance suggests that they possess favorable genetic attributes for maximizing yield production, making them valuable candidates for further evaluation and incorporation into breeding programs aimed at developing high-yielding hybrids. On the basis of GGE distance, the closest to the ideal genotype could also be regarded as desirable genotypes. The most productive genotypes were, G143, G215, G81, G54, G189, G119, G162, G69, G200, G15, G71 and G50 along with yield (YLD), these genotypes also shown better stability and performance for yield components traits like PL, PWP, HSW (Fig. 6 (H): Pattern D). Prioritizing these genotypes in breeding programs for further hybrid testing will contribute to sustainable agriculture by providing farmers with improved hybrids capable of maximizing yield potential across drought environments. Considering the work of [44,45], the optimal genotype is defined as having a high yield and being stable across several test environments. Similarly to Ref. [46], the closer one gets to the ideal genotype, the more desirable it is.

The "which-won-where" analysis is a key component of the GGE biplot for GEI analysis. This biplot identifies mega-environment disparities, determining the best genotypes for each environment and highlighting the ideal genotype with high agronomic performance and stability [46,47]. The which-won-where biplot analysis revealed that certain genotypes were well-adapted to specific environments, confirming the presence of genotype-environment interactions. The identified mega-environments for each agronomic trait allowed us to select the best accessions for that particular trait in each environment, especially those at the corners of the polygons in the biplot. These vertex genotypes were identified as the top performers, indicating their adaptability and superior performance in their respective mega-environments. This suggests that the vertex genotypes were most favored by the environments, making them the most responsive and exceptional in terms of potential yield [48]. However, vertex genotypes with no environment in their sector are undesirable due to their poor performance across different environments [41]. In this study, the genotypes exhibited varying agronomic trait values across environments, indicating crossover genotype-environment interactions (GEIs). Similar findings have been reported by researchers [49,50]. Understanding the environmental adaptation of varieties is crucial for comprehending their genetic basis, which is achieved through analyzing genotype-by-environment interactions [51].

A similarity in bi plot pattern has been observed between the traits -panicle weight per hectare and grain yield per hectare, indicating that lines exhibiting higher stability for panicle weight also demonstrate higher stability for grain yield. The consistent stability patterns in these two traits confirm the possibility of using panicle weight as a proxy for grain yield. This finding is particularly useful in shuttle breeding or when two planting seasons are in close proximity, allowing for efficient selection of genotypes based on panicle weight per hectare, which will yield similar results for grain yield per hectare.

Partial regression coefficients are used to represent the direct effects, they quantify the impact of one variable on other controlling factors. For instance, in Table 5, an increase or decrease in PWt by one standard deviation had approximately ten times the effect on grain yield, as an equivalent shift in plant height by one standard deviation. Grain yield was shown to be correlated with traits other than DFF and MAT, although with varying degrees of intensity and in different directions. The increased MAT indirect effect primarily accounted for the magnitude difference between the direct effect of DFF and the correlation with grain yield, while the discrepancy between the direct effect of MAT and the correlation with grain yield depended on DFF for both magnitude and direction, and on NLP and PWt to a lesser extent. The indirect effects produced by DFF, MAT, and PWt were largely responsible for the disparity between the HSW direct effect and the association with grain yield.

Grain yield was shown to be positively correlated with PL, PW, and PWt via both direct and indirect impacts of PWt. Therefore, the grain yield and the main component attributes were both determined solely by panicle weight in this study. This discovery is crucial in cases involving shuttle breeding, or when two planting seasons occur in close proximity to one another (e.g., kharif *vs.* rabi season in Asia) and not enough manpower or time is available to thresh whole fields and/or weigh grains from extensive breeding pipelines. Here, the breeder may collect panicles from all of the plots, weigh them for selection, and then thresh a subset of them for seeds to grow selected varieties next year. Depending on the breeder's recommendations, the time and energy spent threshing the remaining panicles and/or weighing the seed may be saved and rescheduled for next season or during a slack after the current season is planted. The importance of panicle length on grain yield was also reported by Ref. [52], whereas the importance of panicle length and panicle weight was reported by Ref. [53].

Identifying fully fertile restorers and completely sterile maintainers from the available germplasm is a critical aspect of sorghum breeding projects, particularly in utilizing elite germplasm for hybrid development. Establishing reliable maintainer and restorer lines is a foundational step in hybrid breeding. Following this, evaluating lines with high general combining ability for yield becomes supreme important step of the breeding program. This process not only diversifies nuclear backgrounds but also optimizes the use of identified universal restorer lines in creating high-yielding hybrids. Notably, universal restorers such as ICSV 15014 and ICSR 13009 have demonstrated commendable general combining ability for yield per hectare under irrigated conditions. The identified universal restorer parents exhibiting significant general combining ability and specific combining ability broadens the scope for exploiting alternate cytoplasmic sources in conventional sorghum breeding and in heterosis breeding. This not only enables cytoplasmic diversification but also provides valuable insights into the effects of cytoplasm on various agronomic, phenological, and quality traits under iso-nuclear or alloplasmic backgrounds. Furthermore, the inclusion of identified stable restorer lines in hybrid breeding programs can expedite the development of elite hybrids with improved yield performance. By incorporating these lines into crossing schemes, breeders can accelerate the breeding cycle and ultimately expediting the release of superior cultivars to the market.

Seed producibility of seed parental lines is a fundamental aspect of hybrid breeding, crucial for hybrid development and utilization. Maintainer lines are the backbone of hybrid seed production, ensuring genetic purity and uniformity in the resulting hybrids. Consistent production of high-quality seeds is vital for effectively harnessing hybrid vigor. Seed producibility provides breeders with reliable sources of parent lines, enabling them to develop hybrids with desired agronomic traits such as increased yield and stress tolerance. It also allows breeders to continuously improve hybrid performance by incorporating superior germplasm into various cytoplasmic sources, based on performance in different environments and cytoplasmic backgrounds. This adaptability helps address emerging challenges such as climate change and evolving consumer preferences.Among the studied cytoplasmic sources, A2 cytoplasm had exhibited high seed producibility followed by A1-this diversification of parental lines along with high seed producibility under different environmental conditions, enhances the resilience of hybrids against environmental stresses and market fluctuations. By enhancing the genetic diversity and adaptability, producibility of seed parental lines contributes to long-term food security and agricultural sustainability. By facilitating the production of hybrid seeds tailored to specific agro ecological conditions and consumer preferences, contribute to expanding the range of hybrids available to farmers.

## 5 Conclusions

The study examined the utilization of alternate cytoplasmic backgrounds, identified and established restorers, and ran L × T design to identify superior and stable hybrid products and determine the value of the lines in hybrid combinations. The current study revealed that restoration ability of various restorers on different cytoplasmic backgrounds utilizing GGE biplots and identified that restorer genotypes G10 (ICSR 13009), G4 (IS 33844-5), G8 (ICSV 15014), and G5 (M 35-1-19) are the stable and universal as they showed high restoration ability across the four cytoplasms. The four newly identified restorers were selected as the top best performing genotypes with high stability for restoration, and these genotypes will be useful for the development of drought-tolerant heterotic hybrids with different cytoplasmic backgrounds. They can be utilized to gain insight on the impact of cytoplasm on agronomic performance in both isonuclear and alloplasmic backgrounds. Two (G10 (13009), G8 (ICSV 15014)) of the universal restorers showed significant combining ability (SCA and GCA) for yield under irrigated conditions and across environment (SCA), which enables the breeders to use these restorer lines both in conventional breeding and in heterosis breeding. We observed the following order of cytoplasm's restoration superiority, A_2_ >A_1_>A_3_ >A_4_. The A_2_ cytoplasm can therefore reliably offer seed producibility and seed setting advantage to commercial seed producers. GGE stability model showed that drought had a substantial effect on cultivar performance. A high-yielding cultivar that combines performance per se and stability is a top priority for breeders, researchers, and producers, and is target for scaling out and enhanced adoption in the production environment. Among the 280 genotypes assessed under four environments, the stable and superior hybrid genotypes identified through GGE biplot analyses of ranking genotype were G143, G215, G189, G200, G69, G162, G119, G54, and G81, close to the ideal line. They are the candidates for downstream testing stages for potential release.

## CRediT authorship contribution statement

**Krishna Kasanaboina:** Writing – original draft, Investigation, Formal analysis. **B.V. Vara Prasad:** Writing – review & editing. **Sonal Chavan:** Writing – review & editing. **C.V. Sameer Kumar:** Writing – review & editing. **D. Saida Naik:** Writing – review & editing. **D. Srinivasa Chary:** Writing – review & editing. **Vinod Kumar Reddy Yaram:** Writing – review & editing. **Sunita Gorthy:** Writing – review & editing. **Ephrem Habyarimana:** Writing – review & editing, Supervision, Methodology, Conceptualization.

## Additional information

No additional information is available for this paper.

## Data availability statement

Data will be made available on request.

## Funding statement

This research did not receive any specific grant from funding agencies in the public, commercial, or not-for-profit sectors.

## Declaration of competing interest

All the authors declare that they have no conflict that may have influenced the research presented in this paper.
